# Gamma Knife radiosurgery for cerebral cavernous malformation

**DOI:** 10.1038/s41598-019-56119-1

**Published:** 2019-12-24

**Authors:** Cheng-Chia Lee, Wei-Hsin Wang, Huai-Che Yang, Chung-Jung Lin, Hsiu-Mei Wu, Yen-Yu Lin, Yong-Sin Hu, Ching-Jen Chen, Yu-Wei Chen, Chien-Chen Chou, Yo-Tsen Liu, Wen-Yuh Chung, Cheng-Ying Shiau, Wan-Yuo Guo, David Hung-Chi Pan, Sanford P. C. Hsu

**Affiliations:** 1 Department of Neurosurgery, Neurological Institute, Taipei Veterans General Hospital, Taipei, Taiwan; 2 Department of Radiology, Taipei Veterans General Hospital, Taipei, Taiwan; 3 School of Medicine, National Yang-Ming University, Taipei, Taiwan; 4 Cancer Center, Taipei Veterans General Hospital, Taipei, Taiwan; 5 Department of Neurological Surgery, University of Virginia Health System, Charlottesville, VA USA; 6 Department of Neurosurgery, Shuang Ho Hospital, Taipei Medical University, Taipei, Taiwan; 7 Department of Neurology, Neurological Institute, Taipei Veterans General Hospital, Taipei, Taiwan; 8 Brain Research Center, National Yang‐Ming University, Taipei, Taiwan; 9 Institute of Brain Science, National Yang-Ming University, Taipei, Taiwan

**Keywords:** Diseases of the nervous system, Cerebrovascular disorders, Cerebrovascular disorders

## Abstract

This is a retrospective study examining the efficacy and safety of Gamma Knife radiosurgery (GKS) in treating patients with cerebral cavernous malformations (CCMs). Between 1993 and 2018, 261 patients with 331 symptomatic CCMs were treated by GKS. The median age was 39.9 years and females were predominant (54%). The median volume of CCMs was 3.1 mL. The median margin dose was 11.9 Gy treat to a median isodose level of 59%. Median clinical and imaging follow-up times were 69 and 61 months, respectively. After the initial hemorrhage that led to CCM diagnosis, 136 hemorrhages occurred in the period prior to GKS (annual incidence = 23.6%). After GKS, 15 symptomatic hemorrhages occurred within the first 2 years of follow-up (annual incidence = 3.22%), and 37 symptomatic hemorrhages occurred after the first 2 years of follow-up (annual incidence = 3.16%). Symptomatic radiation-induced complication was encountered in 8 patients (3.1%). Mortality related to GKS occurred in 1 patient (0.4%). In conclusion, GKS decreased the risk of hemorrhage in CCM patients presenting with symptomatic hemorrhage. GKS is a viable alternative treatment option for patients with surgically-inaccessible CCMs or significant medical comorbidities.

## Introduction

Prevention of recurrent hemorrhage and hemorrhage-associated complications are the primary objectives of cerebral cavernous malformation (CCM) treatment. Surgical resection is the definitive treatment for CCMs. However, stereotactic radiosurgery has played an increasingly important role in deep-seated CCMs and in patient with high surgical risks over the past 20 years. In our previous study published in 2005^1^, Gamma Knife radiosurgery (GKS) decreased the annual incidence of hemorrhage in CCMs from 29.2% to 10.3% (within 2 years) and to 3.3% (beyond 2 years) over a mean follow-up period of 5.4 years (range: 9–123 months). Seizure control was achieved in 53% of patients (Engel Class I and II), and there were no treatment-related deaths^1^. GKS also decreased the annual incidence of hemorrhage in brainstem CCMs from 31.3% to 4.29% (within 2 years) and to 3.64% (beyond 2 years)^2^. Therefore, GKS appears to be an alternative treatment for patients with CCMs. In this study, we report the outcomes of 261 CCM patients treated with GKS.

## Results

### Patient population

A consecutive series of 261 patients presenting 331 CCMs underwent GKS between March 1993 and June 2018. The median age was 39.9 years (range: 7.4–75.3 years) and females were predominant (54%). The location of the 331 CMs varied: 111 lesions were found in the brainstem (33.5%), 47 in the basal ganglia and thalamus (14.2%), 115 in the cortical/subcortical region (34.7%), and 41 in the cerebellum (12.4%).

Among the 261 patients, 149 patients (57.1%) had one symptomatic hemorrhage, 99 patients (37.9%) had two symptomatic hemorrhages, 9 patients (3.5%) had three symptomatic hemorrhages, 2 patients (0.8%) had four symptomatic hemorrhages, 1 patient (0.4%) had five symptomatic hemorrhages, and 1 patient (0.4%) had ten symptomatic hemorrhages prior to GKS (Table 1**)**. All patients had signs and symptoms that corresponded to CCMs, such as hemiparesis (47.5%), headache (30.3%), cranial nerve deficits (28.4%), hemisensory deficits (25.7%), dizziness (23.0%), and seizure (13.8%) (Table 1).

**Table 1:** Characteristics of 261 patients with 331 CCMs treated with GKS between 1993 and 2018.

Characteristic	No. (percentage or range)
Age in yrs (range)	39.9 (7.4–75.3)
Gender (% female)	141 (54)
Total no. of CCMs	331
**No. of CCMs by location**	
Brainstem	111 (33.5)
Basal ganglion/thalamus	47 (14.2)
Cortical/subcortical	115 (34.7)
Cerebellum/4^th^ ventricle	41 (12.4)
Multiple lesions	17 (5.1)
**No. of pre-GKS hemorrhages (% of 261 total)**	
1	149 (57.1)
2	99 (37.9)
3	9 (3.5)
4	2 (0.8)
5	1 (0.4)
>5	1 (0.4)
CCM volume (cm^3^)	3.1 (0.03–28.9)
**Symptoms**	
Hemisensory deficit	194 (74.3)
Cranial nerve deficits	187 (71.7)
Hemiparesis	124 (47.5)
Headache	79 (30.3)
Dizziness	60 (23.0)
Seizure	36 (13.8)
Clinical follow-up median (months)	68.9 (6–280)
Image follow-up median (months)	60.7 (6–266)
**GKS Parameters (mean)**	
Margin dose (Gy)	11.9 (8.5–18.0)
Max dose (Gy)	20.3 (10.9–35.0)
Isodose level (%)	59 (50–90)

### Pre-GKS incidence of hemorrhage

We calculated the pre-GKS incidence of hemorrhage among patients that experienced >1 bleeding episode. The pre-GKS observation period extended from the first symptomatic, image-documented hemorrhage to the time of GKS (577.09 patient-years), during which there were 397 hemorrhages. After excluding the initial hemorrhages that led to diagnosis, the calculated annual incidence of hemorrhage was 23.6% (136 hemorrhages/577.09 patient-years).

### Post-GKS incidence of hemorrhage

Hemorrhage episodes can be classified as symptomatic or asymptomatic bleeding. In this study, hemorrhage was defined as any new hemorrhage on MRI with or without neurological symptoms. The post-treatment observation period was the period from the time of GKS until any one of the following: the most recent clinical or imaging follow-up, surgical intervention, or death. The median post-GKS image follow-up time was 60.7 months (range: 6–266 months), with an overall observation period of 1635.08 patient-years. During this period, 130 hemorrhages among 112 patients were observed (0.5 hemorrhages per patient). Among these, 42 hemorrhages occurred within 2 years after GKS, whereas 88 episodes occurred >2 years after GKS. The annual incidence of hemorrhage during the first 2 years after GKS was 9.02% (42 hemorrhages/465.40 patient-year). The annual incidence of hemorrhage after the initial 2-year follow-up was 7.52% (88 hemorrhages/1169.68 patient-year).

**Figure 1:**
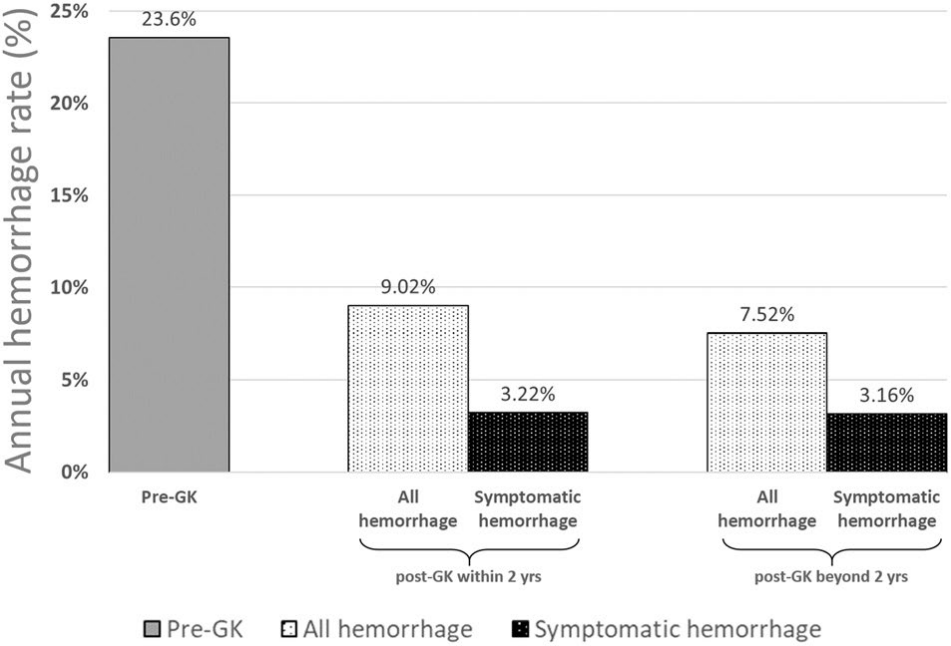
Annual incidence of hemorrhage before and after GKS.

**Figure 2:**
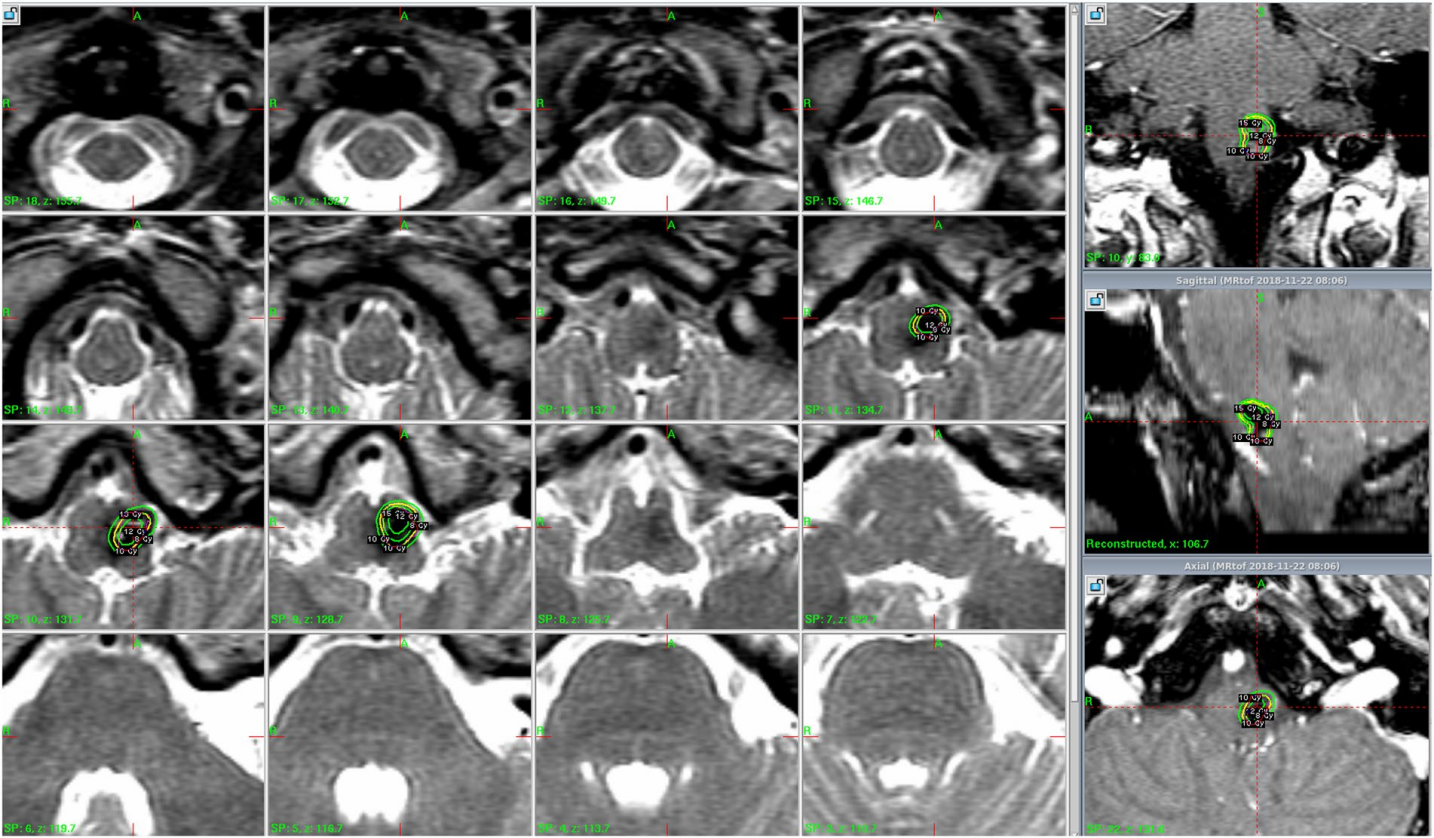
A 27-year-old female presented with a sudden onset of left limb weakness and numbness, gait disturbance, diplopia, and facial numbness for one week. A CCM with associated hemorrhage that measured 3.5 mL in volume was found in the medulla. T2-weighted imaging on MRI demonstrated a hemosiderin ring around the CCM. The CCM was treated using GKS with a margin dose of 10 Gy at a 55% isodose level. The CCM was controlled for up to 63 months.

A total of 52 symptomatic hemorrhages occurred in 43 patients during this period (0.16 symptomatic hemorrhages/patient). Among these, 15 symptomatic hemorrhages occurred within 2 years after GKS, whereas 37 symptomatic hemorrhages occurred >2 years after GKS. The annual incidence of symptomatic hemorrhage during the first 2 years after GKS was 3.22% (15 hemorrhages/465.40 patient-years). The annual incidence of symptomatic hemorrhage after the initial 2-year follow-up was 3.16% (37 hemorrhages/1169.68 patient-years). Figure 1 illustrates the changes in annual incidence of hemorrhage before and after GKS. Figure 2 illustrates a case of a patient with brainstem CCM who presented with symptomatic hemorrhage treated with GKS.

### Seizure control

Prior to GKS, 36 patients with 39 CCMs presented with seizures, and 29 patients had seizures related to CCM hemorrhage. (Table 2) Among the 27 patients with AED-controlled epilepsy, 22 (82%) patients had improvements in seizure control (Engel class I-III). (Table 3) However, 5 (18%) patients had no improvement in seizure control (Engel class IV). Among the 9 patients with drug-resistant epilepsy, only 2 patients had seizure frequency reduction (Engel class II), while the remaining 7 patients had increased seizures (Engel class IV), and 4 patients eventually underwent craniotomy for CCM resection due to poor seizure control. (Table 3) Overall, a total of 28 (78%) patients had improvement in seizure control, and 8 (22%) patients had worsening of seizure control.

**Table 2:** Locations of 39 CCMs in 36 patients who presented with seizures.

Location	No.	Evidence of associated hemorrhage	AED-controlled epilepsy	Drug-resistant epilepsy
Temporal	9	2	2	7
Parietal	13	12	12	1
Frontal	6	6	5	1
Occipital	0	0	0	0
Insula	4	4	4	0
Corpus callosum	2	2	2	0
Corona radiata	2	1	2	0
Other	3	2	3	0
Total	39	29	30*	9*

*30 CCMs in 27 patients without drug-resistant epilepsy, 9 CCMs in 9 patients with drug-resistant epilepsy.

**Table 3:** Seizure outcomes in 27 patients without drug-resistant epilepsy and 9 patients with drug-resistant epilepsy after GKS by Engel classification.

Engel classification	AED-controlled epilepsy (n = 27)	Drug-resistant epilepsy (n = 9)	
	GKS	GKS only	GKS + surgical resection
Class I	4	0	4
Class II	11	2	0
Class III	7	0	0
Class IV	5	3	0
Total	27	5	4

Note that most of the patients with drug-resistant epilepsy had temporal CCMs (n = 7/9, 78%), whereas the other CCMs were parietal or frontal in location. All craniotomies were performed for temporal lobe CCMs (n = 4/4). All 4 of these patients achieved seizure-freedom after CCM resection.

### Adverse radiation effects

New neurological deterioration after GKS without new hemorrhage were found in 8 patients (3.1%). Among them, one patient had cyst formation, and the others developed permanent neurological deficits due to non-hemorrhagic adverse radiation effects. In 16 other patients (6.1%), new T2 signal abnormalities were observed adjacent to their CCMs; however, these patients were neurologically unchanged. Overall, the neurological status after GKS was stable or improved in 96.9% of the patients.

## Discussion

The risk of hemorrhage for a CCM remains undefined^3–5^. Natural history studies^4,6–8^ have suggested that the annual risk of hemorrhage ranges between 2.3% and 4.1%, whereas in surgical series^3,4,9–13^, this risk ranges between 2.7 and 6.8% annually prior to intervention. The risk for recurrent hemorrhage in CCMs is increased after an initial hemorrhage, and this risk can be increased to up to 40%^14–17^. Although other risk factors have been implicated in CCM hemorrhage, predicting when a CCM will hemorrhage remains challenging. Current treatment options for CCMs include observation, microsurgical resection, and radiosurgery. The selection of treatment modality is based mainly on number of hemorrhages, seizure control, and surgical risks. Although radiosurgery may not provide definitive cure for CCM patients, its minimally-invasive nature may provide a safer alternative for patients with surgically-inaccessible CCMs or those with medical comorbidities that preclude surgery. The radiobiological effects of GKS on CCMs remain unclear. However, gradual endothelial cell proliferation and hyalinization yielding luminal closure are two proposed mechanisms. Histological findings of GKS-treated CCMs demonstrated fibrinoid necrosis, endothelial cell destruction, and marked fibrosis in the stroma of connective tissue^18,19^. Thus, the observed decrease in the annual incidence of hemorrhage after GKS may be attributed to the delayed luminal closure of vascular channels.

In this study, the annual incidence of symptomatic hemorrhages was approximately 3% after GKS, which was a dramatic decrease from an annual incidence of 24% prior to GKS. When asymptomatic hemorrhages were included, this incidence was 9% within the first 2 years after GKS and 8% with longer follow-up. AREs were encountered in 3% of the patients. In our prior study of CCM patients with high surgical risks, the incidence of hemorrhage was 10% annually within the first 2 years after GKS, and 3% with longer follow-up^1^. For brainstem CMs, the annual incidence of hemorrhage after GKS was 4% within the first 2 years, and remained approximately 4% thereafter. AREs were observed in 4% of these patients^2^. Up to 2018, there have been three large studies on the use of GKS (>100 cases with at least 4 years of follow-up) specifically for the treatment of repeated hemorrhagic or symptomatic CCMs, comprising a total of 530 patients **(**Table 4)^1,20,21^. The data presented in our study adds to the mounting evidence that GKS decreases the risk of hemorrhage in CCMs. Therefore, GKS may be an option for patients with eloquently located CCMs.

**Table 4:** Literature review: Radiosurgical results of CCMs (>100 cases, follow-up duration > 4 years).

Study, year	Case no.	Margin dose (Gy)	Annual hemorrhage rate (%)			Morbidity (%, radiosurgery-related, AREs)	Mortality (%)
			Pre-GKS	Post-GKS	FU (m)		
Liscak *et al*., 2000	107	16.0	2.0	1.6	48	4.5%	1.9%
Liu *et al*., 2005	125	12.1	29.2	10.3 (<2 yr) → 3.3 (>2 yr)	65	2.4%	0%
Kida *et al*., 2015	298	14.6	21.4	7.4 (<2 yr) → 2.8 (>2 yr)	68	6.7%	2.3%
Present study	261	11.9	23.6	All hemorrhages 9.02% (<2 yr) → 7.52 (>2 yr) Symptomatic hemorrhages 3.22 (<2 yr) → 3.16 (>2 yr)	61	3.1%	0.4%

Abbreviation: AREs: adverse radiation effects, CM: cavernous malformations, GKS: gamma-knife radiosurgery, Gy: gray, m: month, N/A: not available, yr: year

Our institutional approach to treating eloquent CCMs (i.e., brainstem) tends to be more aggressive, even for patients with only one hemorrhage. According to a previous study^22^, the annual incidence of hemorrhage for brainstem CCMs after GKS decreased from 9.5% (within 1 year) to 4.7% (within 2 years). In different study, the annual incidence of hemorrhage after GKS decreased from 15% (within 2 years) to 2.4% (beyond 2 years)^23^. Nagy *et al*. stratified brainstem CCM patients into two groups: low-risk (hemorrhage ≤ 1 episode) and high-risk (hemorrhage ≥ 2 episodes). In the high-risk group, the annual incidence of hemorrhage decreased from 15% (within 2 years after GKS) to 2.4% (beyond 2 years after GKS), and the low-risk group exhibited a decrease from 5.1% (within 2 years) to 2.4% (beyond 2 years)^24^. Radiosurgery-related morbidity ranged from 3.2% to 11.8%. However, Liscak reported transient symptoms in 28% of brainstem CCM patients after GKS^21^. Mortality has not been reported in recent studies^21,22,24^. Radiosurgery for eloquently located CCMs appears to provide good hemorrhage control and may be the treatment of choice for select patients (Table 5).

**Table 5:** Literature review: Radiosurgical results of brainstem CCMs ( >40 cases, follow-up duration > 36 m).

Study, year	Case no.	Margin dose	Annual hemorrhage rate (%)			Morbidity (%, radiosurgery-related, ARE)	Mortality (%)
			Pre-GKS	Post-GKS	FU (m)		
Kida *et al*., 2009	63	13.4	—	9.5 (<1 yr) → 4.7 (1–2 yr)	55	3.2%	—
Monaco *et al*., 2010	68	15.8	32.4	8.2 (<2 yr) → 1.4 (>2 yr)	62	11.8%	0%
Nagy *et al*., 2010	79	12.0	30.0	HR:15(<2 yr) → 2.4 (>2 yr) LR: 5.1(<2 yr) → 1.3 (>2 yr)	48	7.3%	0%
Lee *et al*., 2012	49	11.0	31.3	4.3 (<2 yr) → 3.6 (>2 yr)	41	4.1%	0%
Lee *et al*., 2014	49	12.0	38.4	8.3 (<2 yr) → 1.8 (>2 yr)	64	0%	0%
Liu *et al*., 2016	43	11.9	25.0	3.9 (<2 yr) → 1.9 (>2 yr)	36	2.3%	0%
Lopez *et al*., 2017	95	11.9	3.06	1.4 (<3 yr) → 0.2 (>3 yr)	78	7.4%	0%
Kefeli *et al*., 2018	81	12.0	8.6	0.87	50	4.0%	0%
Park et al., 2018	45	13.0	40.1	3.3 (<2 yr) → 1.48 (<5 y)	112	2.2%	0%
Present study	111	12.0	31.3	3.8 (<2 yr) → 3.1 (>2 yr)	57	5.0%	0%

Abbreviation: ARE: adverse radiation effects, CM: cavernous malformations, GKS: gamma-knife radiosurgery, Gy: gray, m: month, HR: high risk group, LR: low risk group, N/A: not available, yr: year

Seizures are common in patients with CCMs, and there appears to be a correlation between hemorrhage and the seizures. New onset seizures and incremental seizures are often accompanied by recent hemorrhage. Many patients also experienced concomitant headaches or dizziness that may be observed with CCM hemorrhages^22^. Animal studies have suggested that the deposition of blood clot-related metabolites, particularly iron, to be epileptogenic. MRI studies have also suggested that seizures in CCM patients have a temporal relationship to hemorrhages. Other risk factors for seizure development include supratentorial localization, cortical involvement, and archicortical/mesiotemporal localization. Previous studies have reported that approximately 0–18% of patients with infratentorial CCMs suffered from seizures, this was compared to 50–63% of patients with supratentorial CCMs who suffered from seizures^5,8,25,26^. 57–70% of patients with cortical CCMs had seizures, whereas only 14–20% of patients with exclusively subcortical CCMs had seizures^5,8,25^. Differentiation between the cortex and the subcortex may be challenging in older studies that utilized computed tomography as the imaging modality. Recent studies using MRI reported that 49 of 81 CCM patients with cortical involvement suffered from seizures, whereas 0 of 17 CCM patients with exclusively subcortical localization suffered from seizures^26^. CCMs in the temporal lobe are also commonly associated with seizures. One study found seizures in 8 of 9 patients with mesiotemporal CCMs. In contrast, only 41 of 72 patients with other neocortical CCMs suffered from seizures^26^. This suggests that archicortical/mesiotemporal CCMs are associated with a higher incidence of epilepsy^27^. Another study reported similar results in the incidence of mesiotemporal CCMs versus other neocortical CCMs (23.8% versus 3.8%). In our experience, surgical resection is preferred over radiosurgery for temporal CCMs, which are associated with recurrent hemorrhages and drug-resistant epilepsy.

The radiobiological effects of GKS on CMs remains uncertain; however, gradual endothelial cell proliferation and hyalinization yielding luminal closure are two possible mechanisms. Gewirtz *et al*. and Nyáry *et al*. reviewed the histology of patients who underwent GKS^18,19^. Their lesions presented indications of fibrinoid necrosis, endothelial cell destruction, and marked fibrosis in the stroma of connective tissue. This means that the decrease in the annual incidence of hemorrhage after GKS may be due to a delay in the luminal closure of vascular channels.

In the past, the effectiveness of radiosurgery for CCMs was limited by poor neuroimaging (pre-MRI period), excessive radiation doses (>15 Gy), and incomplete or overlarge target coverage. Advancements in neuroimaging, reasonable doses, and better planning software has greatly reduced the risk of complications. Although the definitive treatment for CCM is microsurgical resection, GKS is a viable treatment option for those with surgically-inaccessible CCMs or significant medical comorbidities.

## Methods

### Patient consent and institutional review

A consecutive series of 261 patients presenting 331 CCMs underwent GKS between March 1993 and June 2018. Patient consent was not required by the institutional review board (IRB) committee due to the retrospective nature of the review and because data had been anonymized. (Taipei Veteran General Hospital IRB number: 2018-09-007BC).

The treatment criteria for the GKRS including: A) patient with a cavernoma or multiple cavernomas, B) hemorrhage at least once, C) the hemorrhage causes the clinical symptoms.

### Gamma knife radiosurgery

Radiosurgery was performed using the Leksell Gamma Unit Model C (Elekta Instrument, Inc). The median lesion volume was 3.1 ml (0.03–28.9 ml). Figure 2 presents a typical dose plan for a representative case. The prescription dose was set at an isodose level of 50–90%, and the median margin dose was 11.9 Gy (range 8.5–18 Gy). A higher margin dose (>12 Gy) was avoided due to the benign nature of the lesions and used only in the early part of this study. A higher margin dose (>12 Gy) was avoided due to the benign nature of the lesions and used only in the early part of this stud To achieve a highly conformal dose distribution, multiple small shots were used to maximize the mean dose and minimize the radiation volume outside the target. No identifiable portion of the facial nerve received more than 13 Gy and the trigeminal nerve received no more than 15 Gy. The margin dose administered to tumors that bulged into tissue of the brainstem was reduced to 11 Gy. y. To achieve a highly conformal dose distribution, multiple small shots were used to maximize the mean dose and minimize the radiation volume outside the target. No identifiable portion of the facial nerve received more than 13 Gy and the trigeminal nerve received no more than 15 Gy. The margin dose administered to tumors that bulged into tissue of the brainstem was reduced to 11 Gy.

### Follow-up imaging and clinical evaluation

Following GKS, all of the patients underwent MR imaging studies and clinical evaluation at 6-month intervals. The median follow-up time was 60.7 months (range 6–266 months). We divided those patients into two groups based on their follow-up time: 171 patients were followed up regularly for at least 2 years, and another 90 patients had regular follow-up for less than 2 years. The patients were carefully examined for any clues of hemorrhage, including new foci of high signal intensity in T1WI, volume expansion of radiated lesions, or edematous changes in T2WI. Not all hemorrhages were symptomatic; however, we recorded the data for further analysis.

It is not uncommon for anti-epileptic drugs (AEDs) to be taken for at least 2 years after GKS for seizure control in our protocol of treating cavernoma-related epilepsy (CRE). Prior to GKS, 36 patients with 39 CCMs presented with seizure, including 30 patients with AEDs control and 9 patients with drug-resistant epilepsy (Table 2). The definition of drug-resistant epilepsy is the failure of two antiepileptic trials that were tolerated, chosen appropriately, and administered (either individually or together) in order to achieve a seizure-free state^28^. Engel’s classification was used to evaluate the effectiveness of GKS in seizure control^29^.

## Data Availability

All data generated or analysed during this study are included in this published article.
